# Photosynthetic and secondary metabolic responses of biological soil crusts to combined warming, reduced precipitation, and enhanced UV-B radiation: divergent strategies across developmental stages

**DOI:** 10.3389/fpls.2026.1930890

**Published:** 2026-09-09

**Authors:** Yalin Zhou, Rong Hui, Shuanglang Li

**Affiliations:** 1 Shapotou National Field Observation and Research Station for Desert Ecosystem, Northwest Institute of Eco‐Environment and Resources, Chinese Academy of Sciences, Lanzhou, China; 2 College of Life Sciences, University of Chinese Academy of Sciences, Beijing, China

**Keywords:** biological soil crusts, combined stress, photosynthetic physiology, secondary metabolites, UV-B radiation

## Abstract

**Introduction:**

Biological soil crusts (BSCs) are key components of dryland ecosystems, yet their physiological stability under concurrent changes in UV-B radiation, temperature, and precipitation remains poorly understood. In particular, how BSCs at different developmental stages regulate carbon acquisition and secondary metabolism under multifactor climate stress remains unclear.

**Methods:**

In this study, we investigated the physiological and metabolic responses of algal, lichen, and moss crusts exposed for two years to factorial combinations of warming and reduced precipitation (+1.5 °C and -8%) and UV-B enhancement (ambient, +18%, +24%) in open-top chambers (OTCs). We quantified photosynthetic traits (chlorophyll a, chlorophyll b, and gross primary productivity) and secondary metabolites (flavonoids, total phenolics, carotenoids, and extracellular polysaccharides).

**Results:**

The results revealed that UV-B radiation was the dominant driver of physiological and metabolic variation in BSCs, consistently suppressing photosynthetic performance and secondary metabolite accumulation across all BSC types. The strongest inhibition occurred in algal crusts, indicating high sensitivity but limited physiological plasticity. In contrast, lichen and moss crusts exhibited greater regulatory capacity and partial recovery under combined warming and reduced precipitation, suggesting stress-buffered metabolic adjustment. Notably, several physiological and metabolic traits exhibited partial recovery under combined stress treatments relative to the UV-B-only treatments, suggesting that altered temperature and moisture conditions may modulate the effects of UV-B through physiological buffering rather than yielding simple additive effects.

**Discussion:**

This study reveals the divergent adaptation strategies of BSCs at different developmental stages under combined climatic stresses. The results highlight the importance of incorporating successional-stage heterogeneity and physiological trade-offs into predictions of dryland ecosystem responses under future climate change.

## 1 Introduction

Global climate change is markedly altering the environmental conditions of dryland ecosystems. As greenhouse gas emissions continue to increase, global temperatures are rising, precipitation regimes are shifting, and extreme climatic events are becoming more frequent (IPCC, 2023). In drylands, water availability is a key factor limiting ecosystem structure and function. Warming can increase atmospheric evaporative demand, but its effects on actual evapotranspiration and soil water deficit depend on soil moisture availability and local hydroclimatic conditions. Reduced precipitation or altered distributions of seasonal precipitation may further reduce soil water availability and intensify drought stress (He et al., 2022). Consequently, arid and semi-arid ecosystems are generally highly sensitive to climate change (Stringer et al., 2022). In the arid regions of northwestern China, warming is projected to continue under future climate change, and some areas may experience reduced precipitation, intensified aridity, or altered precipitation patterns (Qin et al., 2014; Goyal et al., 2019).

UV-B radiation is an important environmental factor that regulates surface ecological processes in drylands. Although the Montreal Protocol has promoted the gradual recovery of the stratospheric ozone layer, the rate and magnitude of ozone recovery vary across regions and remain subject to long-term climatic and atmospheric uncertainties. Moreover, surface UV-B radiation is not determined by stratospheric ozone alone but is also jointly regulated by cloud cover, aerosols, water vapor, surface albedo, altitude, and seasonal changes in solar radiation (Bernhard et al., 2023; Chipperfield and Bekki, 2024; Neale et al., 2025). Therefore, even under a general trend of ozone recovery, surface UV-B exposure may remain high or increase episodically in some regions (Liaqat et al., 2023; Neale et al., 2025). In drylands, sparse vegetation cover, extensive bare-soil exposure, low atmospheric humidity, and intense solar radiation can further amplify the ecological relevance of UV-B stress. Changes in UV-B radiation often occur concurrently with warming and altered precipitation regimes, thereby jointly affecting the physiological activity, community structure, and ecological functions of surface biological communities.

Biological soil crusts (BSCs) are specialized biotically active complexes formed by cyanobacteria, lichens, mosses, fungi, and their hyphae and exudates binding with soil particles (West, 1990; Weber et al., 2022). They are widely distributed in arid, semi-arid, and other fragile ecosystems worldwide (Belnap et al., 2001). As important surface components of desert ecosystems, BSCs play critical roles in stabilizing the soil surface, reducing wind and water erosion, promoting carbon and nitrogen cycling, and improving microhabitats for plant establishment (Rodriguez-Caballero et al., 2022; Weber et al., 2022). The formation and development of BSCs display a clear successional pattern, typically beginning with early-stage algal crusts dominated by pioneer cyanobacteria and progressively transitioning to lichen crusts and moss crusts (Chen et al., 2024). Across developmental stages, algal crusts generally exhibit high tolerance to extreme environments and can survive and colonize under drought, intense radiation, and nutrient-poor conditions. Lichen crusts tend to depend on relatively stable habitats and grow slowly, whereas moss crusts are more commonly distributed in moist and shaded microsites and have stricter requirements for soil texture and moisture conditions (Ju et al., 2021; Sun and Li, 2022). BSCs at different developmental stages differ markedly in terms of their morphological structure, water-holding capacity, nutrient accumulation, and responses to environmental stress, suggesting that their sensitivity and adaptation to external environmental changes may be strongly stage dependent (Li et al., 2021).

Under external stresses such as UV-B radiation, warming, and water deficit, BSCs may alleviate photosystem damage and oxidative stress through physiological regulation of photosynthesis and secondary metabolic responses, thereby helping maintain physiological stability under stress (Hui et al., 2015; Ladrón de Guevara and Maestre, 2022; Kashi Zenouzi et al., 2024). Chlorophyll content and gross primary productivity (GPP) reflect the light-harvesting capacity and carbon assimilation performance of BSCs, respectively, and can be used to assess photosynthetic responses to environmental stress (Kuroda, 2023; Qiu et al., 2024). Previous studies have shown that warming and reduced precipitation alter the water use efficiency, metabolic activity, and community structure of BSCs (Guan et al., 2025; Qiu et al., 2026), whereas UV-B radiation can directly affect the pigment system, photosynthetic processes, and carbon fixation capacity of phototrophic organisms (Dwivedi and Ahmad, 2023). Additionally, BSCs can regulate secondary metabolites such as carotenoids (Cars), flavonoids, total phenolics, and extracellular polysaccharides (EPSs) to increase UV-B screening, reactive oxygen species (ROS) scavenging, and microhabitat buffering capacity (Kumari et al., 2021; Pagels et al., 2021). However, most existing studies have focused on the effects of single climatic factors on the physiological and ecological characteristics of BSCs (Hui et al., 2016; Raggio et al., 2023; Rubio Gomez-Roso et al., 2025), whereas temperature, moisture, and UV-B radiation often change synchronously under natural conditions. Warming and reduced precipitation may alter the physiological state of BSCs and thereby modify their sensitivity to UV-B radiation. However, whether these stressors amplify or offset each other under realistic climate change scenarios and whether such interactions differ across developmental stages remain unclear. Moreover, although photosynthetic carbon acquisition and secondary metabolism are closely linked processes underlying stress adaptation, whether carbon limitation caused by reduced photosynthetic performance constrains investment in protective metabolites under multifactor stress remains unknown.

Therefore, we hypothesized that enhanced UV-B radiation, together with warming and reduced precipitation, would generate nonadditive effects on the physiological and metabolic processes of BSCs by altering carbon allocation between assimilation and defense. We further hypothesized that BSCs at different developmental stages would exhibit contrasting resilience strategies because of differences in structural complexity and metabolic regulation. To test these hypotheses, we exposed algal, lichen, and moss crusts from the Shapotou region of the Tengger Desert to factorial combinations of warming, reduced precipitation, and enhanced UV-B radiation. We quantified photosynthetic traits and secondary metabolites and integrated physiological measurements with multivariate analyses to identify stage-dependent strategies underlying BSC responses to future climate change.

## 2 Materials and methods

### 2.1 Study area

This study was conducted in an artificial sand-fixing vegetation area located north of the Baotou–Lanzhou Railway at the Shapotou Desert Research and Experimental Station, Zhongwei city, Ningxia Hui Autonomous Region, China (37°27′ N, 104°57′ E; elevation 1250 m). The region has a temperate continental climate characterized by hot and relatively wet summers and cold, dry winters. The mean annual temperature is 9.6 °C, the mean annual precipitation is 186 mm, which is concentrated mainly from June to August, and the annual potential evaporation is approximately 3000 mm. Local climate monitoring over the past decade reveals that the regional mean annual temperature increased by approximately 0.5 °C, whereas the mean annual precipitation decreased by approximately 4 mm. These changes indicate a local warming–drying tendency in the Shapotou region, which is consistent with the reported warming trend in arid regions of China and precipitation variability in Ningxia (Li et al., 2016; Yang et al., 2020; Wang et al., 2022). This area is located on the southeastern fringe of the Tengger Desert and supports BSCs at different developmental stages (Lan et al., 2012), making it a suitable site for studying the physiological and biochemical responses of BSCs to enhanced UV-B radiation under climate change. The algal crusts are dominated by cyanobacteria, with dominant species including *Microcoleus vaginatus* and *Nostoc carneum* (Hu et al., 2003); the lichen crusts are dominated by *Collema coccophorum*, *Collema tenax*, and *Endocarpon pusillum* (Li, 2012); and the moss crusts are dominated by *Bryum argenteum*, *Didymodon vinealis*, and *Syntrichia caninervis* (Wan et al., 2025).

### 2.2 Sample collection

Well-developed algal, lichen, and moss crusts were randomly collected from the experimental area. For each BSC type, 18 intact samples, including the underlying native sand substrate, were collected using square polyethylene trays (16 × 16 × 5 cm) with drainage holes at the bottom. To maintain the structural integrity of the BSCs and their underlying substrate and to minimize disturbance-induced sand coverage during sampling, all the samples were uniformly pre-wetted before collection. Distilled water was slowly and evenly added to the surface of each BSC sample until the crust layer was fully moistened and water had infiltrated into the underlying surface sand, thereby enhancing cohesion between the BSCs and the substrate. After sufficient water infiltration and structural stabilization, the intact BSC samples were collected together with the underlying sand substrate. This procedure was applied consistently across all BSC types and subsequent treatment groups to reduce sampling disturbance and preserve sample integrity; therefore, it was not considered an independent experimental factor. After collection, loose surface sand and external debris were gently removed with a soft brush to minimize potential interference from surface sand cover in subsequent measurements.

### 2.3 Experimental design

Field-collected BSC samples were randomly placed on the global change experimental platform at the Shapotou Desert Research Station. A set of open-top chambers (OTCs), constructed with glass walls and aluminum alloy frames (Li et al., 2018, Li et al., 2023), with a single side dimension of 100 × 150 cm, was used to simulate warming and altered precipitation conditions. The OTCs passively increased air temperature by transmitting shortwave solar radiation while reducing wind exchange. In addition, wind-driven rainfall interception on chamber walls resulted in reduced effective precipitation inputs compared with ambient conditions. Control plots were established at least 2 m away from the OTCs to minimize experimental interference. Long-term microclimatic monitoring indicated the effects of OTCs on air temperature, precipitation, humidity, and photosynthetically active radiation (PAR). The effects of OTCs on warming coupled with the reduction in rainfall are described in detail in **Table 1**. Compared with the control plots, the OTCs increased the mean annual temperature by 1.5 °C and reduced the mean annual precipitation by 8% (Li et al., 2018). Because these two environmental changes were generated simultaneously by the OTCs, their individual effects could not be statistically separated in this experiment. Although the OTCs slightly reduced light transmittance, no significant differences in photosynthetically active radiation (PAR) were observed among treatments; therefore, the effect of light variation was considered negligible (Li et al., 2018).

**Table 1:** Effects of the size of the open-top chamber (OTC) on annual warming, rainfall reduction, and humidity and photosynthetically active radiation (PAR) (mean ± SE) based on meteorological observations at the Shapotou Desert Research and Experiment Station, Chinese Academy of Science (SDRES, CAS).

Treatment	Annual temperature (°C)	Annual precipitation (mm)	Relative humidity (%)	PAR (μmol m^-2^ s^-1^)
Inside OTC	12.8 ± 2.7	153.7 ± 19.9	43.6 ± 6.6	229.0 ± 59.3
Outside OTC	11.3 ± 1.8	167.7 ± 22.1	45.8 ± 6.8	312.0 ± 23.0

To investigate the effects of UV-B radiation under these warming and altered precipitation conditions, a UV-B supplementation experiment was conducted in combination with the OTC system. Previous studies have shown that atmospheric ozone concentrations in the mid-latitudes of the Northern Hemisphere have decreased by approximately 6–9% compared with historical levels and have remained relatively low over subsequent decades. In addition, a 1% reduction in stratospheric ozone concentration can result in an approximately 2% increase in surface UV-B radiation flux (Solomon, 1999). Although reductions in the emissions of ozone-depleting substances are expected to promote ozone recovery, the restoration of stratospheric ozone concentrations to historical levels will require several decades, and elevated UV-B exposure is therefore likely to persist in the near future. Accordingly, on the basis of the average summer UV-B irradiance in the Shapotou region, three UV-B levels were established (2.75, 3.25, and 3.41 W m^-2^), corresponding to ambient conditions and increases of 18% and 24%, respectively. Considering the local background UV-B environment, the two enhanced levels were designed to represent moderate and relatively high-UV-B enhancement scenarios, with the goal of evaluating the sensitivity of BSCs on exposed dryland surfaces to increasing UV-B radiation. UV-B radiation was applied using a square-wave irradiation regime.

UV-B radiation was generated using narrowband UV-B fluorescent lamps (Philips TL 20W/01, 20 W), with peak emission centered at 311 nm. To eliminate UV-C radiation, each lamp was wrapped with a 0.13 mm cellulose acetate filter, which cut off wavelengths below 280 nm. The cellulose acetate filters were replaced every 5 days to ensure stable spectral quality and consistent UV-B transmission throughout the experiment. The lamps were suspended above the biological soil crusts, and the UV-B irradiance was regulated by adjusting the distance between the lamps and the crust surface. UV-B radiation slightly decreased because of OTC material attenuation, and all the UV-B treatments were therefore corrected on the basis of the measured transmission coefficient to achieve the intended enhancement levels using a UV radiometer (Photoelectric Instrument Factory of Beijing Normal University, China). This setup ensured a stable and ecologically relevant UV-B radiation regime throughout the experimental period. The 2.75 W m^-2^ treatment was designed as a structural control, with identical lamp-supporting frames installed but without UV-B lamps or cellulose acetate filters. UV-B exposure was conducted for 8 h daily (9:00–17:00) and was maintained continuously throughout the experimental period, except under rainy or overcast conditions.

**Figure 1:**
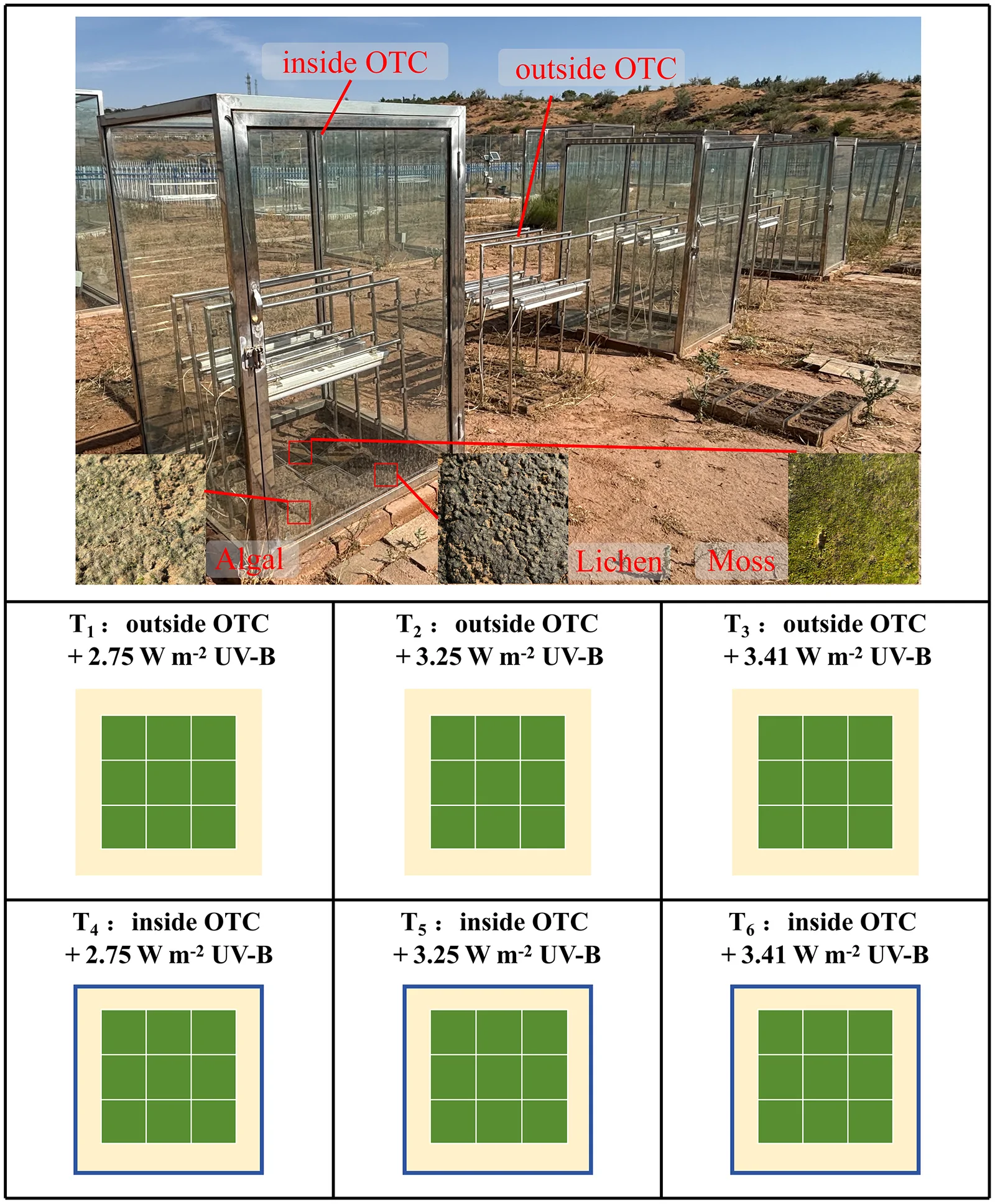
Field experimental setup and treatment design for combined warming and UV-B radiation stress.

A fully factorial experimental design was adopted to disentangle the independent and interactive effects of microclimatic modification and UV-B irradiance on BSCs. Six experimental treatments were established as follows (**Figure 1**): outside the OTC with an ambient UV-B irradiance of 2.75 W m^-2^ (T_1_); outside the OTC with a UV-B irradiance elevated by 18% relative to ambient conditions, corresponding to a radiation intensity of 3.25 W m^-2^ (T_2_); and outside the OTC with a UV-B irradiance elevated by 24% relative to ambient conditions, corresponding to a radiation intensity of 3.41 W m^-2^ (T_3_). The three complementary treatments inside the OTC included an ambient UV-B irradiance of 2.75 W m^-2^ (T_4_); a UV-B irradiance elevated by 18% relative to ambient conditions, corresponding to a radiation intensity of 3.25 W m^-2^ (T_5_); and a UV-B irradiance elevated by 24% relative to ambient conditions, corresponding to a radiation intensity of 3.41 W m^-2^ (T_6_). This design enabled a direct comparison between ambient field conditions and microclimate-altered conditions induced by the OTCs, as well as a gradient analysis of UV-B radiation intensity under both environments. Within each treatment plot, three types of biological soil crusts (algal crusts, lichen crusts, and moss crusts) were introduced. For each crust type, three independent subsamples (n=3) were randomly distributed within each plot. All the samples were arranged in a fully randomized spatial pattern to minimize microsite heterogeneity and positional bias.

### 2.4 Physiological and biochemical measurements

After two years of exposure to the treatments, surface BSC samples were collected using a stainless-steel core cutter (inner diameter of 1.3 cm) and transported to the laboratory for determination of chlorophyll a (Chl a), chlorophyll b (Chl b), carotenoids (Car), flavonoids, total phenolics, and extracellular polysaccharides (EPS). Considering the small-scale spatial heterogeneity within BSC communities, each biological sample was collected using a five-point random sampling method. The subsamples collected from the five sampling locations within each sample were thoroughly homogenized to obtain a representative sample. For each combination of treatment and BSC type, three independent biological replicates were obtained. This sampling strategy reduced the influence of microsite variability and minimized the contribution of localized extreme values, thereby improving the representativeness and stability of each biological replicate. In addition, the gross primary productivity (GPP) of the BSCs under each treatment was measured *in situ*.

Chlorophyll and Car contents were determined by the anhydrous ethanol method (Hui et al., 2016). Fresh samples of equal area were immersed in 25 mL of anhydrous ethanol, ground in an ice bath for pigment extraction, and centrifuged twice at 12000 × g for 30 min each time. The absorbance of the extract was measured with a spectrophotometer at 470, 649, and 665 nm. The Chl a, Chl b, and Car contents were calculated using the following formulas:


$$Chl\;a=13.95A_{665}-6.88A_{649}$$

$$Chl\;b=24.96{\text{A}}_{649}-7.32{\text{A}}_{665}$$

$$Car=\left(1000{\text{A}}_{470}-2.05\text{ Chl a}-114\text{ Chl b}\right)/245$$

X=CVn/S

where X (mg cm^−2^) is the pigment content, C (mg L^−1^) is the pigment concentration, V (L) is the extraction volume, n is the dilution factor, and S (cm^2^) is the sampling area.

GPP was measured using a static chamber method coupled with a Li-6400 portable photosynthesis system (LI-COR, Inc., USA). Measurements were conducted on clear days between 09:00 and 11:00 under relatively stable environmental conditions, with air temperatures maintained between 20 and 28 °C and adequate PAR levels to support active photosynthesis. After the change in CO_2_ concentration stabilized, CO_2_ concentrations were recorded at 10 consecutive time points at equal intervals to calculate the CO_2_ exchange rate. The CO_2_ exchange rates measured under light and dark conditions were used to estimate the net CO_2_ exchange rate (NEE) and ecosystem respiration rate (ER), respectively, of the BSCs. The GPP was calculated using the following formula:


$$Fc=\left(dc^{\prime }/dt\right)\left(1000-W\right)VP/RST$$

GPP=ER−NEE

where Fc (μmol m^−2^ s^−1^) is the CO_2_ exchange rate, GPP (μmol m^-2^ s^-1^) is the gross primary productivity of the measured plants, dc′/dt is the water-vapor-corrected rate of change in the CO_2_ concentration, V (m^3^) is the chamber volume, P (kPa) is the mean chamber air pressure during measurement, W (kPa) is the water vapor partial pressure in the chamber during measurement, R (8.314 J mol^−1^ K^−1^) is the gas constant, S (m^2^) is the chamber base area, and T (K) is the temperature inside the chamber during measurement.

The contents of flavonoids, total phenolics, and EPS were quantified by the aluminum nitrate, Folin–Ciocalteu and phenol–sulfuric acid colorimetric methods (Khaleghnezhad et al., 2019; Shraim et al., 2021; Chowaniec et al., 2025), respectively. For flavonoid and total phenolic extraction, equal-area fresh BSC samples were oven-dried at 40 °C, ground into powder, and extracted with 70% ethanol under dark conditions at 40 °C. The extracts were obtained after repeated extraction and centrifugation (4000 rpm, 15 min, 4 °C), and the combined supernatants were adjusted to a final volume for subsequent determination. EPS was extracted from fresh samples by homogenization in ice-cold water, followed by ethanol precipitation and centrifugation. The obtained extracts were subjected to colorimetric analysis, and the absorbance values were measured at 510, 765, and 490 nm for flavonoids, total phenolics, and EPS, respectively. Quantification was performed using calibration curves generated from rutin, gallic acid, and glucose standards. The contents were calculated using the following formula:


X=CVn/S

where X (mg cm^−2^) represents the contents of flavonoids, total phenols and EPS; C (mg mL^−1^) represents the concentration of the target index obtained from the standard calibration curve; V (mL) represents the final volume of the extract after dilution to volume; n (cm^2^) represents the dilution factor; and S represents the sampling area.

### 2.5 Statistical analysis

All the data were organized in Microsoft Excel and analyzed using IBM SPSS Statistics 26.0 and R 4.2.3. Prior to statistical analysis, the data were checked for outliers and tested for normality using the Shapiro–Wilk test and for homogeneity of variance using Levene’s test. When necessary, logarithmic, square-root, or Box–Cox transformations were applied to improve normality and variance homogeneity. Variables that still failed to satisfy the assumptions of parametric ANOVA after transformation were analyzed using the aligned rank transform (ART). In this study, Car failed to meet the assumptions of normality and homogeneity of variance even after multiple transformations. Accordingly, ART-ANOVA was used for the analysis of Car. EPS satisfied the prerequisites for parametric tests following logarithmic transformation; thus, three-way ANOVA was used to analyze EPS together with the other indicators (**Supplementary Table 1**). Three-way ANOVA was used to test the main and interactive effects of BSC type (Type), UV-B radiation level (UV-B), warming and reduced precipitation treatment (WD), and their interactions on each physiological and metabolic indicator. When significant interaction effects were detected, simple effects were further analyzed. The six combined UV-B × WD treatments were compared separately within each BSC type using one-way ANOVA followed by Tukey’s HSD test for multiple pairwise comparisons among treatment means. Different lowercase letters in the figures indicate significant differences among treatments within the same BSC type at P< 0.05. For variables without significant three-way interactions, these within-type comparisons were used to display treatment response patterns, whereas biological interpretation was based primarily on the significant main effects or lower-order interactions identified by three-way ANOVA. Prior to principal component analysis (PCA) and redundancy analysis (RDA), all the variables were subjected to Z score transformation to ensure comparability among the variables with different units. PCA was used to evaluate overall variation in photosynthetic physiological and secondary metabolic traits among BSC types and treatments. An RDA was used to examine the relationships between the response variables and experimental factors. The significance of the RDA axes and explanatory variables was assessed using permutation tests.

## 3 Results

### 3.1 Effects of combined stress on the photosynthetic performance of BSCs at different developmental stages

**Figure 2:**
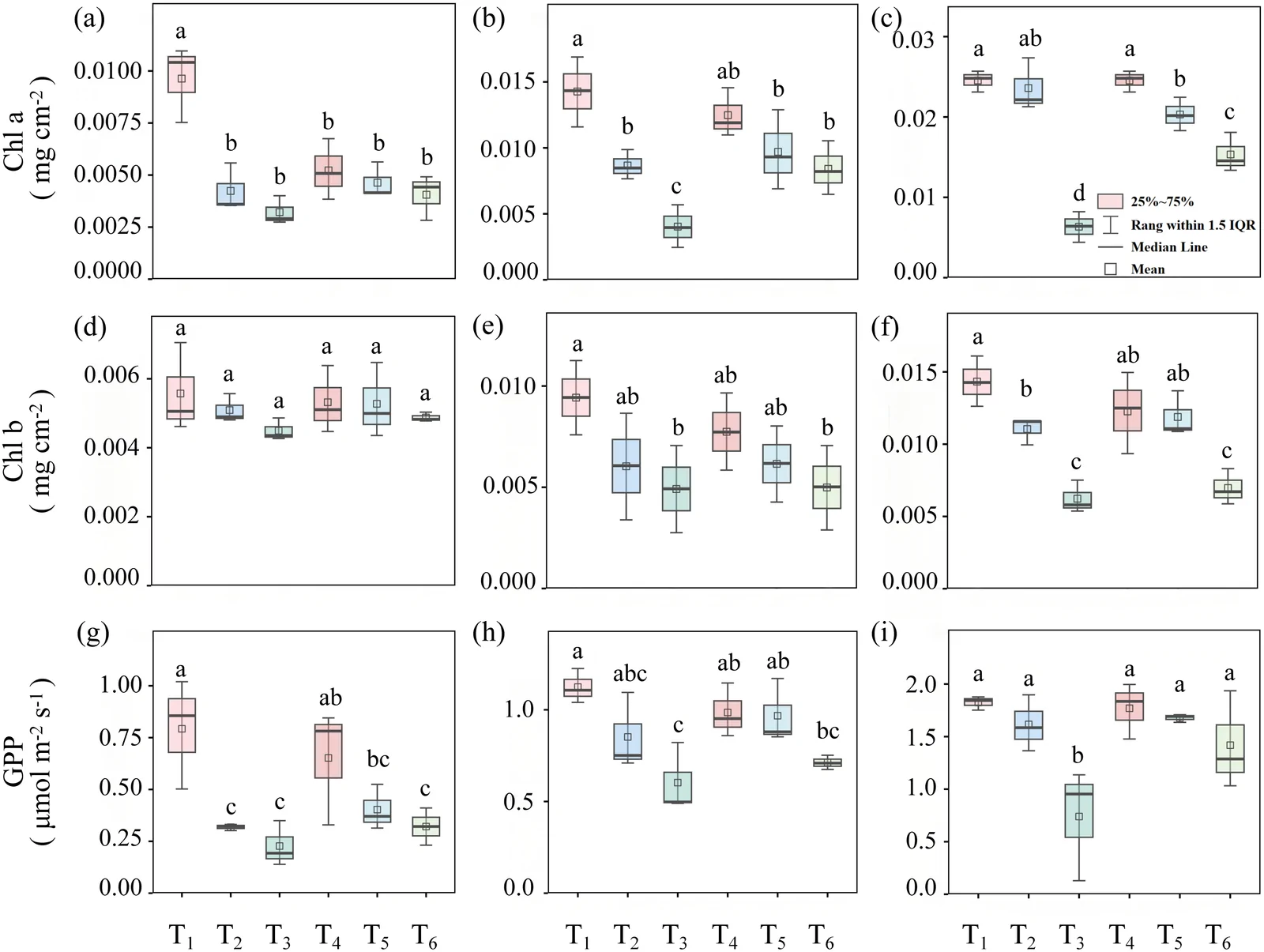
Responses of the photosynthetic physiology of BSCs at different developmental stages to stress. Panels **(a, d, g)** represent the Chl a content, Chl b content, and GPP of the algal crusts, respectively; Panels **(b, e, h)** represent the Chl a content, Chl b content, and GPP of the lichen crusts; and Panels **(c, f, i)** represent the Chl a content, Chl b content, and GPP of the moss crusts, respectively. T_1_-T_6_: outside OTC + 2.75 W m^−2^ UV-B (T_1_), outside OTC + 3.25 W m^−2^ UV-B (T_2_), outside OTC + 3.41 W m^−2^ UV-B (T_3_), inside OTC + 2.75 W m^−2^ UV-B (T_4_), inside OTC + 3.25 W m^−2^ UV-B (T_5_), inside OTC + 3.41 W m^−2^ UV-B (T_6_). Different lowercase letters indicate significant differences among treatments at P< 0.05.

The Chl a content was significantly influenced by BSC type (F = 252.427; P< 0.001; ηp^2^ = 0.93; **Table 2**), UV-B radiation (F = 85.140; P< 0.001; ηp^2^ = 0.83; **Table 2**), and their interaction with WD (Type × UV-B × WD: F = 4.119; P< 0.01; ηp^2^ = 0.31; **Table 2**). These results indicated that BSC developmental stage was the dominant factor explaining variation in Chl a content. Under UV-B-only treatments, Chl a consistently decreased across all crust types. Relative to the control (T_1_), algal crusts showed reductions of 56.00% and 66.60% under T_2_ and T_3_, respectively, while lichen crusts decreased by 39.27% and 71.74%, and moss crusts declined by 74.17% under T_3_ (P< 0.05; **Figures 2a–c**). Under OTC-induced warming and reduced precipitation conditions, Chl a remained negatively affected by increasing UV-B, but partial recovery was observed compared with UV-B-only treatments; notably, lichen and moss crusts exhibited significant increases of 52.04% and 58.65% under T_6_ compared with T_3_ (P< 0.05; **Figures 2b, c**).

**Table 2:** Three-way ANOVA results for the effects of biological soil crust type, UV-B radiation, and warming and reduced precipitation on photosynthetic and secondary metabolic traits.

Treatments	Effect df	Chl a		Chl b		GPP		Flavonoid		Total phenolic		Car		EPS	
		F	ηp^2^	F	ηp^2^	F	ηp^2^	F	ηp^2^	F	ηp^2^	F	ηp^2^	F	ηp^2^
Type	2	252.427***	0.93	52.036***	0.74	93.575***	0.84	150.110***	0.89	120.878***	0.87	133.057***	0.88	721.205***	0.98
UV-B	2	85.140***	0.83	23.483***	0.57	22.702***	0.56	162.924***	0.90	351.296***	0.95	78.485***	0.81	299.998***	0.94
WD	1	1.741^ns^	0.05	0.175^ns^	0.01	2.019^ns^	0.05	202.226***	0.85	12.771**	0.26	0.015^ns^	<0.01	245.364***	0.87
Type×UV-B	4	15.389***	0.63	5.929***	0.40	1.931^ns^	0.18	26.479***	0.75	36.204***	0.80	25.162***	0.74	37.313***	0.81
Type×WD	2	2.984^ns^	0.14	0.153^ns^	0.01	1.202^ns^	0.06	48.060***	0.75	8.803***	0.33	3.140^ns^	0.15	11.766***	0.40
UV-B×WD	2	15.964***	0.47	1.681^ns^	0.09	3.404*	0.16	112.071***	0.86	44.229***	0.71	8.704***	0.33	173.740***	0.91
Type×UV-B×WD	4	4.119**	0.31	0.256^ns^	0.03	0.977^ns^	0.10	27.930***	0.76	4.319**	0.32	1.096^ns^	0.11	17.729***	0.66

The table presents degrees of freedom (df), F values, and partial eta squared (ηp^2^) for the main effects and interactions of biological soil crust type (Type), UV-B radiation (UV-B), and warming and reduced precipitation (WD). The df represents the numerator degrees of freedom for each tested effect. Values of partial eta squared (ηp^2^)< 0.01 are reported as<0.01. Asterisks indicate significance levels (*P< 0.05, **P< 0.01, and ***P< 0.001), and ns indicates no significant effect.

The Chl b content showed a less pronounced response than the Chl a content, whereas the three-way interaction did not reach statistical significance. The Chl b content was significantly affected by BSC type and UV-B radiation (Type: F = 52.04, P< 0.001; UV-B: F = 23.48, P< 0.001; **Table 2**), with a significant interaction between Type and UV-B radiation (F = 5.93, P< 0.001; **Table 2**). Increased UV-B radiation resulted in a clear decrease in the Chl b content in the moss crusts, whereas the algal and lichen crusts exhibited relatively limited variation among UV-B treatments. OTC-induced warming and reduced precipitation presented weak positive effects on the Chl b content across BSC types, although these differences were not statistically significant.

GPP was significantly influenced by BSC type (ηp^2^ = 0.84; **Table 2**), UV-B radiation (ηp^2^ = 0.56; **Table 2**), and the UV-B × WD interaction (F = 3.40; P< 0.05; ηp^2^ = 0.16; **Table 2**). The significant interaction suggested that OTC-induced warming and reduced precipitation conditions modified the inhibitory effects of enhanced UV-B radiation on carbon assimilation. In the UV-B-only treatments, the GPP generally decreased with increasing UV-B radiation intensity. Compared with that in T1, the GPP in algal crusts decreased by 59.82% and 71.33% under T_2_ and T_3_, respectively, whereas the GPP in lichen and moss crusts decreased by 46.36% and 59.45%, respectively, under T_3_ (P < 0.05; **Figures 2g–i**). Under OTC-induced warming and reduced precipitation, the GPP still decreased with increasing UV-B, but the decrease was smaller than that under the UV-B-only treatments. Notably, the GPP in the moss crust under T_6_ was significantly greater than that under T_3_ by 47.85% (P < 0.05; **Figure 2i**).

### 3.2 Effects of combined stress on secondary metabolites in BSCs at different developmental stages

**Figure 3:**
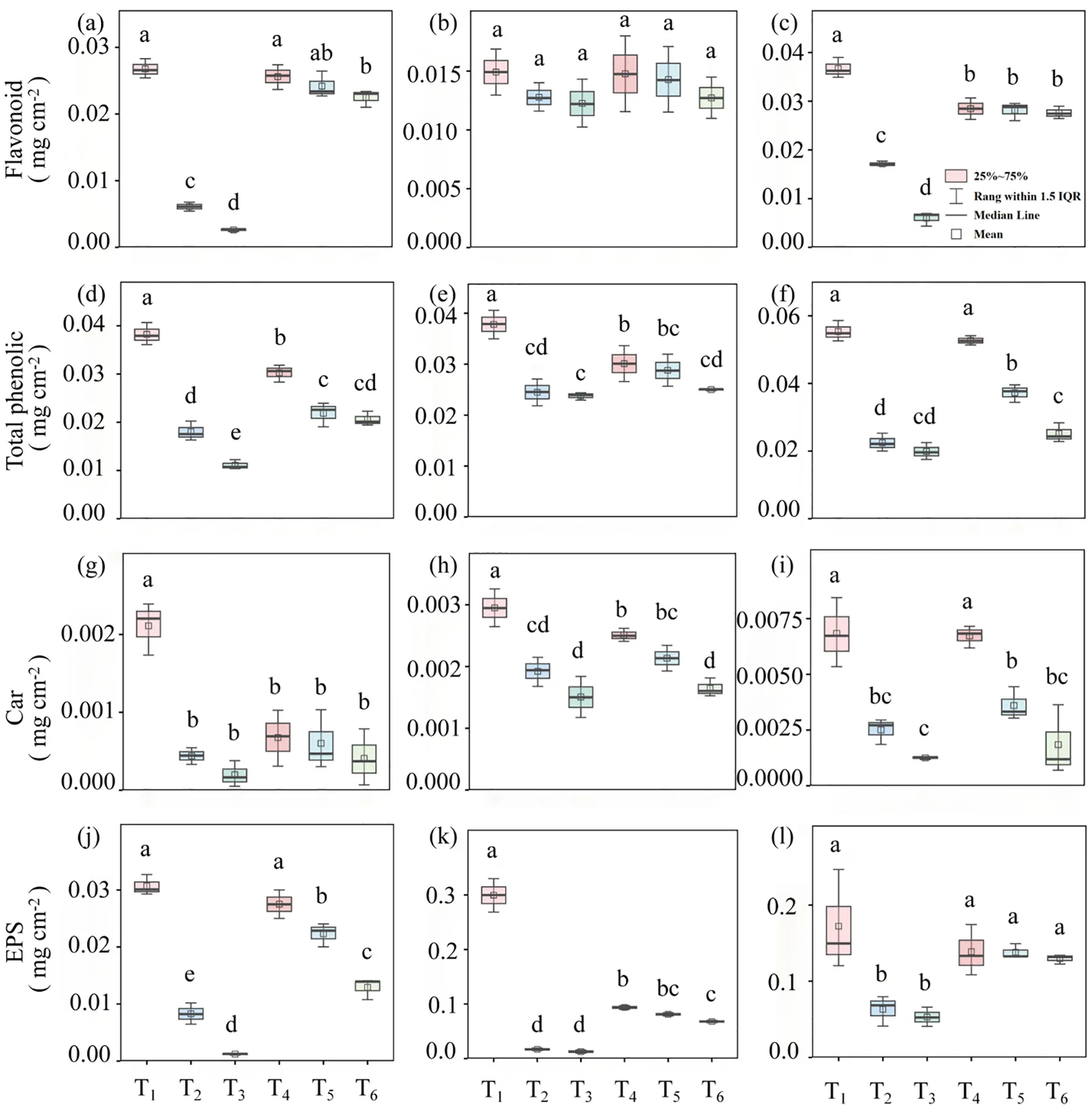
Responses of secondary metabolism in BSCs at different developmental stages to stress. Panels **(a, d, g, j)** represent the flavonoid, total phenolic, Car, and EPS contents of the algal crusts, respectively; Panels **(b, e, h, k)** represent the flavonoid, total phenolic, Car, and EPS contents of the lichen crusts; and Panels **(c, f, i, l)** represent the flavonoid, total phenolic, Car, and EPS contents of the moss crusts, respectively. T_1_–T_6_: outside OTC + 2.75 W m^-2^ UV-B (T_1_), outside OTC + 3.25 W m^-2^ UV-B (T_2_), outside OTC + 3.41 W m^-2^ UV-B (T_3_), inside OTC + 2.75 W m^-2^ UV-B (T_4_), inside OTC + 3.25 W m^-2^ UV-B (T_5_), inside OTC + 3.41 W m^-2^ UV-B (T_6_). Different lowercase letters indicate significant differences among treatments at P< 0.05.

In addition to affecting photosynthetic processes, enhanced UV-B radiation influenced secondary metabolite accumulation in BSCs, with distinct responses among developmental stages and climate treatments. Flavonoid content was significantly affected by BSC type, UV-B radiation, WD, and their interactions (P < 0.001; **Table 2**). Effect size analysis revealed that UV-B radiation (ηp^2^ = 0.90), BSC type (ηp^2^ = 0.89; **Table 2**), and WD (ηp^2^ = 0.85; **Table 2**) accounted for substantial proportions of the variation in flavonoid content. Flavonoid accumulation was greatest in T_1_ and decreased under enhanced UV-B. The most pronounced decreases occurred under T_2_, with decreases of 90.23% and 83.68% in algal and moss crusts, respectively (P< 0.05; **Figures 3a, c**). Under OTC-induced warming and reduced precipitation combined with UV-B, the flavonoid content partially recovered in algal and moss crusts relative to that under the UV-B-only treatments, whereas the flavonoid content of lichen crusts did not significantly differ among the treatments.

The total phenolic content exhibited significant main and interaction effects on BSC type, UV-B, and WD (P< 0.01; **Table 2**). Effect size analysis revealed that UV-B radiation accounted for the greatest proportion of variance in total phenolic content (ηp^2^ = 0.95; **Table 2**), followed by BSC type (ηp^2^ = 0.87; **Table 2**) and the UV-B × WD interaction (ηp^2^ = 0.71; **Table 2**). Under the UV-B-only treatments, the total phenolic content was greatest in T_1_ and generally decreased with increasing UV-B. Compared with that in T_1_, the total phenolic content in the algal crusts in T3 decreased by 70.91%, while that in the lichen and moss crusts was significantly lower under both T_2_ and T_3_ (P < 0.05; **Figures 3d–f**). Under OTC-induced warming and reduced precipitation, the total phenolic content decreased with increasing UV-B intensity in the algal and moss crusts, whereas lichen crusts showed relatively limited variation. Compared with the corresponding UV-B-only treatments, the algal and moss crusts showed partial increases under T_5_ and T_6_.

The Car content was significantly affected by the interactions of UV-B radiation with crust type and with warming combined with reduced precipitation (P< 0.001; **Table 2**), whereas the three-way interaction was not significant (F = 1.10; P = 0.373; **Table 2**). Under the UV-B-only treatments, the Car content was significantly lower than that under T_1_ for all the BSC types, with the Car contents in the algal, lichen, and moss crusts decreasing by 90.61%, 48.96%, and 81.70%, respectively, under T_3_ (P < 0.05; **Figures 3g–i**). Under OTC-induced warming, reduced precipitation and increased UV-B, the Car content in algal crusts remained low and did not differ significantly among treatments, whereas lichen and moss crusts had higher values under T_4_ than under T_5_ and T_6_. Compared with the corresponding UV-B-only treatments, the Car content in lichen and moss crusts was greater under T_5_ and T_6_.

EPS, an important extracellular structural component associated with surface stabilization and water retention, showed strong sensitivity to combined climate stress. EPS content was significantly affected by BSC type, UV-B radiation, and all interaction terms (P< 0.001; **Table 2**). Effect size analysis revealed that BSC type (ηp^2^ = 0.98; **Table 2**), UV-B radiation (ηp^2^ = 0.94; **Table 2**), WD (ηp^2^ = 0.87; **Table 2**), and their interactions accounted for substantial proportions of the variation in EPS content. Under the UV-B-only treatments, the EPS content of all three BSC types was greatest under T_1_ and generally decreased with increasing UV-B radiation. The decline in algal crusts was most pronounced, with a significant decrease of 95.71% under T_3_ relative to T_1_ (P < 0.05; **Figure 3j**). Lichen and moss crusts showed smaller but significant decreases under T_2_ and T_3_ (P < 0.05; **Figures 3k, l**). Under OTC-induced warming and reduced precipitation, the EPS content in algal crusts decreased significantly with increasing UV-B levels (P < 0.05; **Figure 3j**), whereas the changes in lichen and moss crusts were smaller. Compared with that in the corresponding UV-B-only treatments, the EPS content increased under T_5_ and T_6_ in all three BSC types, especially in the algal crusts.

### 3.3 Developmental-stage differentiation in the physiological strategies and environmental responses of BSCs

**Figure 4:**
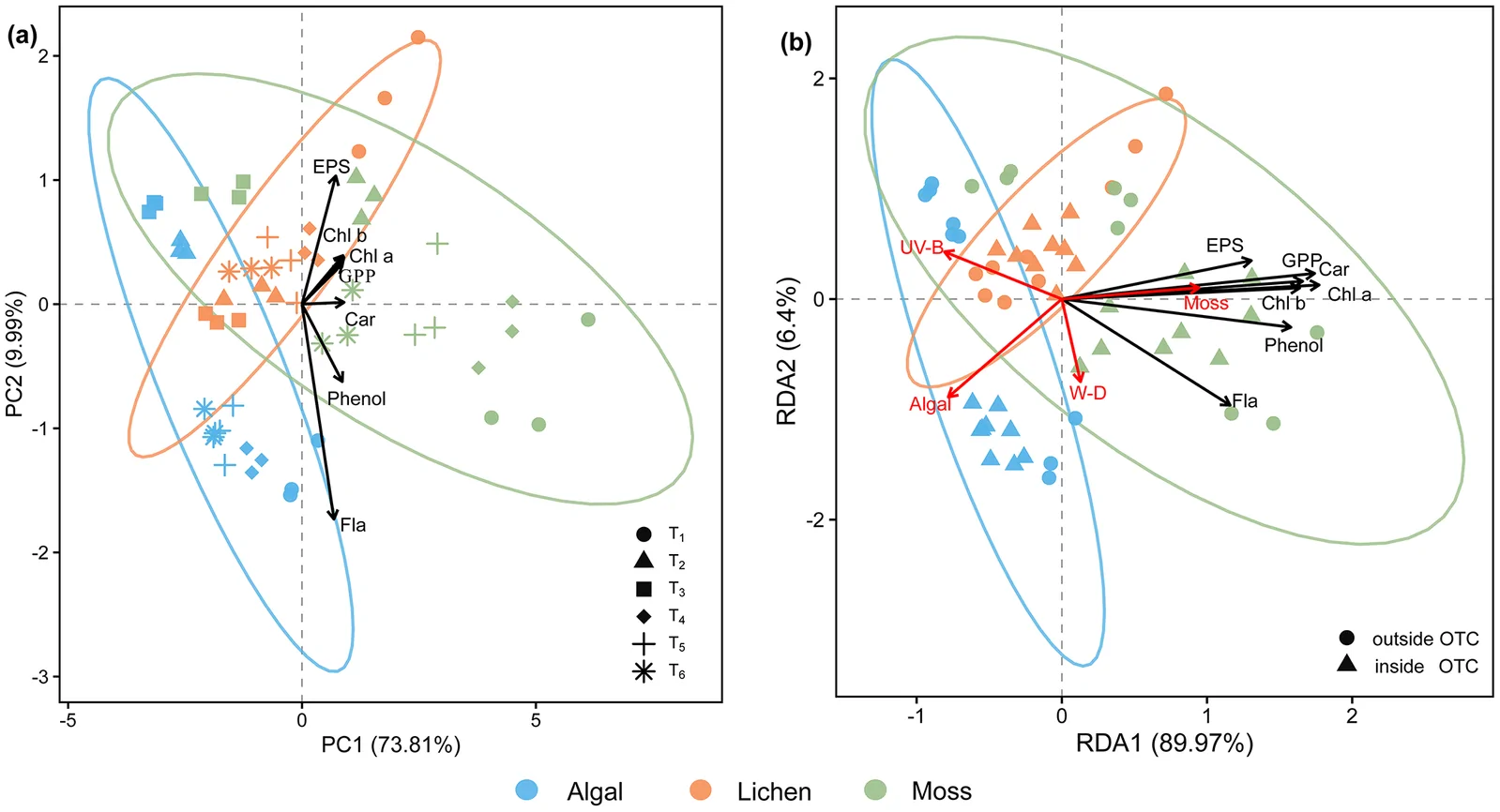
PCA and RDA ordination of physiological and metabolic indicators in BSCs under UV-B and WD treatments. **(a)** Principal component analysis (PCA) showing the overall differentiation pattern of the physiological and metabolic indicators of different BSC types under different treatments; **(b)** redundancy analysis (RDA) illustrating correlations among UV-B radiation, WD, BSC types, and BSC physiological and metabolic indicators. Different colors denote crust categories. In Panel **(a)**, distinct symbols correspond to treatments T_1_-T_6_; in Panel **(b)**, the symbols distinguish between the ambient and WD treatments. The ellipses outline the sample distribution ranges for each crust type. The black arrows denote the response variables Chl a, Chl b, GPP, Car, flavonoids (Fla), total phenolics (Phenol), and EPS; the red arrows represent the explanatory variables UV-B radiation, W-D (warming and reduced precipitation), and crust type (Algal, Lichen, Moss).

Multivariate analyses revealed clear differences in physiological and metabolic strategies among BSC developmental stages under combined climate stress. The PCA results revealed that PC1 and PC2 explained 73.81% and 9.99% of the variance, respectively, with a cumulative explained variance of 83.80% (**Figure 4a**). The samples showed clear separation among the BSC types. Algal crusts were distributed mainly on the negative side of PC1, whereas moss crusts were located mostly on the positive side of PC1, and lichen crusts were distributed between algal and moss crusts but tended to occupy the upper region of the ordination space. This separation indicates distinct physiological and metabolic characteristics among developmental stages. The loading vectors revealed that Chl a, Chl b, Car, GPP, and EPS were positively associated with PC1 and were closely clustered, suggesting that PC1 represented mainly a gradient related to photosynthetic pigment accumulation, photosynthetic productivity, and extracellular polysaccharide production. Moss crusts were positioned closer to these functional traits, whereas algal crusts were associated with lower levels of photosynthetic and metabolic indicators. In contrast, flavonoids and total phenolics contributed mainly to the variation along PC2, suggesting that PC2 represented an independent defense-related metabolic dimension. Although this axis explained a relatively small proportion of the total variance (9.99%), it reflected ecologically meaningful differences in secondary metabolic profiles among BSC developmental stages. Flavonoids and phenolic compounds are key components of UV-B acclimation responses and function as UV-screening compounds and antioxidants that mitigate oxidative damage induced by enhanced radiation. Therefore, variation along PC2 may represent differences in chemical defense capacity rather than general physiological performance.

RDA further revealed the major environmental drivers shaping physiological and metabolic differentiation among BSC types. The first two RDA axes explained 96.37% of the constrained variation, with RDA1 and RDA2 accounting for 89.97% and 6.40%, respectively (**Figure 4b**). The overall RDA model was significant (F = 26.87, P = 0.001), and UV-B radiation had the strongest effect (F = 39.53, P = 0.001), followed by crust type (F = 32.12, P = 0.001) and WD treatment (F = 3.71, P = 0.028). The RDA ordination provided further evidence for the associations between environmental factors, BSC types, and physiological indicators. The vectors of Chl a, Chl b, Car, GPP, and EPS were closely aligned and pointed toward the positive direction of RDA1, whereas the UV-B radiation vector pointed in the opposite direction. The RDA ordination revealed that UV-B radiation was negatively associated with Chl a, Chl b, Car, GPP, and EPS, which is consistent with their reductions under the enhanced UV-B treatments. In contrast, the WD vector was directed mainly toward the negative side of RDA2 and showed a relatively weak association with the major photosynthetic indicators, suggesting that warming and reduced precipitation had weaker regulatory effects on the physiological and metabolic characteristics of BSCs than UV-B radiation did. The crust type vectors also corresponded well with the distribution of sample groups: algal crusts were associated with the negative side of RDA1, moss crusts were closely related to the positive side of RDA1 and to higher photosynthetic and metabolic indicators, and lichen crusts occupied an intermediate position but partly overlapped with moss crusts. The RDA ordination pattern further confirmed the separation among BSC types and revealed that UV-B radiation, warming and reduced precipitation, and crust type jointly shaped the physiological and metabolic differentiation of BSCs, with UV-B radiation and crust type playing dominant roles.

## 4 Discussion

### 4.1 Enhanced UV-B radiation disrupts the balance between carbon acquisition and secondary metabolism in BSCs

Enhanced UV-B radiation simultaneously impaired photosynthetic carbon acquisition and reduced the accumulation of secondary metabolites in BSCs across different developmental stages. This coordinated decline suggests that prolonged UV-B stress may constrain the ability of BSCs to maintain both carbon assimilation and defense-related metabolic investment. These results highlight the close coupling between photosynthetic performance and stress protection under intensified radiation conditions. Although PC1 accounted for the majority of the variation, representing differences in photosynthetic performance and carbon acquisition capacity, PC2 revealed an independent defense-related metabolic dimension among BSC developmental stages. The loading contributions of flavonoids and total phenolics to PC2 indicate that secondary metabolites constitute a distinct component of UV-B acclimation and stress adaptation.

Enhanced UV-B radiation can affect photosynthetic processes through both direct photodamage and oxidative stress pathways. UV-B photons can directly damage the D1 protein of the PSII reaction center, disrupt the integrity of the electron transport chain, and induce the formation of pyrimidine dimers in DNA, thereby impairing genetic information processing (Singh P. et al., 2023). In addition, UV-B radiation can indirectly trigger the overproduction of reactive oxygen species (ROS), leading to membrane lipid peroxidation and protein oxidative degradation (Xue et al., 2022). In this study, the reductions in Chl a and Chl b contents under the enhanced UV-B treatments indicate that light-harvesting capacity and pigment-based photoprotection were compromised, which was further reflected by decreased GPP. Photosynthetic pigments provide the material basis for light reactions, and their decline may weaken the light capture, transfer, and conversion efficiency of BSCs, ultimately limiting community-level carbon assimilation capacity (Simkin et al., 2022; Hoellrich et al., 2023).

The effects of UV-B radiation on BSCs were not limited to the photosynthetic system; their internal metabolic networks also showed corresponding changes, thereby altering the synthesis and accumulation of secondary metabolites (Erb and Kliebenstein, 2020; Jan et al., 2021). Importantly, these metabolites do not represent a homogeneous functional group but rather exhibit distinct ecological roles and response strategies. Flavonoids and total phenolics primarily function as UV screening and antioxidant compounds and are directly involved in scavenging ROS and mitigating radiation-induced oxidative damage (Agati et al., 2012; Bulut et al., 2025). Car contributes primarily to photoprotection by regulating excess energy dissipation and stabilizing the photosynthetic apparatus under high light stress (Dong et al., 2021; Gómez-Sagasti et al., 2023). EPS contributes to extracellular matrix formation, soil particle stabilization, and water retention, thereby enhancing the environmental buffering capacity of BSCs (Irankhahi et al., 2024; Zenouzi et al., 2024). Previous studies have shown that moderate UV-B stress can induce phenylpropanoid metabolism and promote the accumulation of flavonoids and other antioxidant compounds, thereby increasing organismal resistance to radiation stress (Xue et al., 2024; Tian et al., 2024). However, in the present study, the prolonged and enhanced UV-B treatments resulted in an overall decline in these protective traits. These findings suggest that under relatively strong or sustained UV-B stress, the induction capacity of secondary metabolism may become insufficient to compensate for radiation-induced physiological impairment.

The simultaneous reduction in photosynthetic performance and protective metabolites may reflect a shift in resource allocation under prolonged UV-B stress. Enhanced UV-B radiation can impair photosynthetic pigments and photosynthetic performance, thereby reducing the carbon assimilation capacity and potentially tightening the overall carbon budget of BSCs. Under such carbon- and energy-limited conditions, organisms are likely to prioritize basal metabolism, cellular repair, and essential structural maintenance while downregulating carbon-demanding metabolic pathways (He et al., 2022; Xie et al., 2022). The biosynthesis of flavonoids and phenolic compounds requires carbon flux diversion into the phenylpropanoid pathway (Singh V. K. et al., 2023; Wu et al., 2025), whereas EPS represents an important carbon-containing structural pool involved in soil aggregation, water retention, nutrient retention, and biofilm formation (Mota et al., 2021; Chowaniec et al., 2025). Therefore, when UV-B-induced photosynthetic impairment substantially limits carbon acquisition, the metabolic cost of continuously increasing flavonoid, total phenolic, and EPS production may exceed the physiological capacity of BSCs (Hoellrich et al., 2023; Singh et al., 2024; Mazur and Ślesak, 2025), resulting in a simultaneous decline in photosynthetic performance and the production of defense-related metabolites. Similarly, Sun et al. (2010) reported that the flavonoid content in *Ginkgo biloba* leaves changed from increasing to decreasing after prolonged UV-B exposure, and Ri et al. (2024) reported that moderate UV-B exposure can transiently stimulate the accumulation of phenolic compounds, whereas prolonged UV-B stress reduces photosynthetic capacity, photosynthetic pigments, carotenoids, and several secondary metabolites. This coupling suggests that under strong or prolonged UV-B stress, BSCs may be unable to compensate for photosystem damage solely by enhancing secondary metabolism, resulting in a coordinated decline in photosynthetic function and defense-related traits.

### 4.2 Differential sensitivity of BSCs at different developmental stages to stress factors

The three BSC types displayed markedly stage-dependent divergent responses to combined treatments of elevated UV-B radiation, warming and reduced precipitation; these differences can be attributed to the variations in species assemblage, structural complexity, resource acquisition strategies, and physiological and metabolic regulation patterns among BSC types across developmental stages (Qiu et al., 2026). In this study, BSC type significantly affected all the measured physiological and metabolic indicators, and its interactions with UV-B radiation were significant for most variables, including Chl a, Chl b, flavonoids, total phenolics, Car, and EPS. These findings indicate that algal, lichen and moss crusts do not respond uniformly to the same stress regime but instead exhibit distinct physiological and metabolic response strategies associated with their developmental stages.

Algal crusts represent the earliest developmental stage of BSC succession and showed the strongest sensitivity to UV-B radiation in the ordination analysis. In the PCA and RDA ordinations, the algal crusts were distributed mainly in the negative direction of PC1/RDA1 and were associated with relatively low photosynthetic and metabolic trait values. Under the enhanced UV-B treatments, the algal crusts exhibited pronounced reductions in Chl a, GPP, flavonoids, total phenolics, Car, and EPS, suggesting substantial impairment of both carbon acquisition and secondary metabolism. However, this sensitivity does not necessarily indicate poor environmental adaptation. Algal crusts are pioneer communities that frequently experience intense solar radiation, desiccation, and nutrient limitation in desert environments. They possess constitutive protective mechanisms, including extracellular polysaccharides, UV-screening compounds such as scytonemin and mycosporine-like amino acids (MAAs), and efficient antioxidant systems (Hui et al., 2021; Bataeva and Grigoryan, 2024; Chen et al., 2024). Nevertheless, their relatively simple community structure and limited physiological complexity may restrict further metabolic adjustment when UV-B stress exceeds their tolerance threshold. Therefore, algal crusts may exhibit strong environmental tolerance but relatively limited physiological plasticity under intensified climate stress.

Lichen crusts displayed an intermediate response pattern between those of algal and moss crusts, which is consistent with their transitional position in BSC succession. Compared with algal crusts, lichen crusts generally maintained higher levels of Chl a, GPP, and several secondary metabolites under multiple stress treatments, although strong UV-B radiation still exerted a pronounced inhibitory effect. The symbiotic organization of lichens, involving coordinated interactions between photobionts and mycobionts, may provide greater physiological integration and buffering capacity against environmental fluctuations (Stanton et al., 2023). However, this buffering capacity also has an upper limit when radiation stress intensifies.

Moss crusts represent a later successional stage of BSC development and presented markedly greater resistance and recovery capacity than other BSCs. In the PCA and RDA ordination plots, the moss crust samples were distributed mainly along the positive direction of PC1/RDA1 and were closely associated with Chl a, Chl b, Car, GPP, and EPS, reflecting their greater photosynthetic capacity and greater investment in structural substances. Although UV-B radiation significantly reduced several physiological and metabolic indicators in moss crusts, the magnitude of the decline was generally smaller, and recovery under the combined treatments was more evident. These results demonstrate that late-stage crusts improve the ability to balance growth, repair and defense under combined environmental pressures by modulating resource allocation, triggering repair pathways and remodeling metabolic networks (Stanton et al., 2023).

The contrasting responses among BSC developmental stages indicate different strategies for coping with climate stress. Early-stage algal crusts rely primarily on constitutive protective traits but have a limited capacity for further physiological adjustment, whereas later-stage lichen and moss crusts possess greater regulatory flexibility that allows them to maintain photosynthetic activity and secondary metabolism under fluctuating environmental conditions. These stage-dependent strategies highlight the importance of considering successional heterogeneity when predicting BSC responses to future climate change.

### 4.3 OTC-induced warming and reduced precipitation modulate UV-B responses through physiological buffering

In arid regions, BSCs are typically exposed to multiple concurrent stressors, including enhanced radiation, elevated temperature, and water limitation. In this study, some photosynthetic and secondary metabolic indicators were higher under the combined warming, reduced precipitation, and enhanced UV-B radiation treatments than under the corresponding UV-B-only treatments. However, these indicators generally remained markedly lower than those in the control treatment. This pattern suggests that the effects of combined stress on BSCs were nonadditive and may have involved antagonistic or buffering interactions. These findings are consistent with recent evidence showing that the combined effects of UV-B radiation and drought are often nonadditive and may involve cross-resistance or priming responses (Jansen et al., 2022; Shoaib et al., 2024).

Such effects may be related to changes in the physiological state of BSCs under combined stress. Under enhanced UV-B radiation alone, BSCs may remain in a relatively active photosynthetic and metabolic state, making the photosynthetic apparatus more directly exposed to radiation damage and oxidative pressure. Under warming and reduced precipitation, moderate warming may increase metabolic turnover and repair efficiency, thereby partially alleviating UV-B-induced inhibition of the photosynthetic system and protective metabolic pathways (Yan et al., 2024). Alternatively, reduced surface water availability may induce a low-activity or dormant state, reducing the exposure of the photosynthetic system to intense radiation stress (Ekwealor et al., 2021). Therefore, the partial recovery of some indicators under combined stress does not necessarily imply that warming and reduced precipitation directly alleviated UV-B damage. Instead, it may reflect complex physiological regulation under multiple stressors.

The relative contribution of these two mechanisms likely depends on both the UV-B intensity and the developmental stage of a BSC. Under the moderate-UV-B enhancement treatment, warming-induced stimulation of repair and acclimation processes may have contributed strongly to the partial maintenance of physiological function, especially in lichen and moss crusts. These later successional crusts have more complex community organization, greater structural development, and stronger physiological regulatory capacity, potentially enabling them to better maintain pigment pools, metabolic activity, and photoprotective or repair processes when temperature conditions remain within a physiologically favorable range (Veres et al., 2022; Yin et al., 2023; Amitrano et al., 2025). In contrast, under the stronger UV-B enhancement treatment, the capacity of active repair mechanisms may have become increasingly constrained as radiation-induced oxidative and photosynthetic damage accumulated, while the concurrent reduction in water availability likely imposed an additional limitation on physiological recovery and antioxidant regulation (Zhang et al., 2021; Dias et al., 2023). Under these conditions, metabolic downregulation or partial dormancy may play a greater protective role by reducing photosynthetic excitation pressure and limiting further oxidative injury.

The balance between these mechanisms also differed among crust types. The recovery of the photosynthetic and metabolic traits of the algal crusts was relatively limited under the combined treatments, suggesting that the warming-induced increase in active repair was insufficient to compensate for the UV-B-induced damage. As early successional communities with relatively simple structural organization and limited physiological plasticity, algal crusts may depend more strongly on constitutive tolerance and stress-avoidance strategies, including desiccation-induced metabolic suppression. However, this response may protect cellular integrity at the cost of reduced carbon assimilation and secondary metabolite production. In contrast, lichen and moss crusts exhibited greater partial recovery under the combined treatments, particularly for several photosynthetic and metabolic indicators. Their more complex symbiotic or multicellular organization and greater capacity for physiological regulation may facilitate active acclimation, resource reallocation, and repair processes under moderate combined stress. Therefore, active metabolic buffering may be relatively important in lichen and moss crusts, whereas activity suppression and stress avoidance may contribute more strongly to the response of algal crusts, especially under high UV-B intensity and low water availability.

The RDA results further revealed that UV-B radiation was the dominant factor driving the physiological and metabolic differentiation of BSCs, with the strongest explanatory effect, whereas warming and reduced precipitation, as well as BSC type, played important regulatory roles. These findings suggest that under global change, UV-B radiation may be a major driver of BSC physiological responses, whereas temperature and moisture conditions can modulate the sensitivity thresholds of BSCs to UV-B radiation by altering their physiological state, inducing cross-tolerance, or reshaping resource allocation.

### 4.4 Ecological implications of stage-dependent BSC resilience under future climate change

As key biological components of dryland surface ecosystems, BSCs provide essential ecosystem services, including soil stabilization, carbon and nitrogen cycling, water retention, and microhabitat improvement (Yu et al., 2024a; Witzgall et al., 2024). Our study revealed that enhanced UV-B radiation can simultaneously affect photosynthetic carbon assimilation and secondary metabolic processes in BSCs, whereas OTC-induced warming and reduced precipitation may further alter the direction and magnitude of these responses. These findings suggest that the effects of future climate change on BSCs depend not only on the magnitude of change in individual environmental factors but also on interactions among multiple climatic factors. Decreases in photosynthetic pigments and GPP may weaken the carbon fixation capacity of BSCs (Qiu et al., 2024), whereas declines in protective compounds may reduce their capacity for environmental buffering and surface stabilization (Yu et al., 2024b). Such changes could influence the persistence and ecological functions of BSCs in dryland ecosystems under future climate scenarios.

Importantly, the contrasting responses among developmental stages suggest that BSC communities may maintain ecosystem stability through functional differentiation rather than through a uniform stress response. Early-stage algal crusts exhibit strong constitutive adaptation to harsh environments but limited physiological plasticity under intensified stress, whereas later-stage lichen and moss crusts possess greater regulatory flexibility and recovery potential. These stage-dependent strategies may influence the trajectory of BSC succession and ecosystem functioning under changing climatic conditions.

Our results highlight the necessity of incorporating successional heterogeneity and multifactorial climate interactions into future assessments of BSC responses. Because warming, altered precipitation regimes, and changes in UV-B radiation frequently occur simultaneously, responses inferred from single-factor experiments may underestimate or misrepresent the complexity of BSC adaptation. Future studies combining long-term field observations, multifactor manipulation experiments, and process-based modeling will be essential for improving predictions of BSC dynamics and their contributions to dryland ecosystem stability. Furthermore, investigations across broader climatic gradients and different BSC communities are needed to determine the generality of these stage-dependent response strategies.

## 5 Conclusions

Under the experimental conditions of this study, increased UV-B radiation simultaneously constrained the photosynthetic physiology and secondary metabolism of BSCs. Under 24% greater UV-B radiation than under ambient conditions, the GPP in the algal crusts decreased by 71.33%, while the flavonoid, Car, and EPS contents decreased by more than 90%. These results suggest that intensified UV-B stress may disrupt the balance between carbon acquisition and defense-related metabolic investment.

The combined effects of warming, reduced precipitation, and increased UV-B radiation exhibited partially nonadditive response patterns. Several photosynthetic and metabolic indicators showed partial recovery under the combined treatments compared with the corresponding UV-B-only treatments, suggesting that altered temperature and moisture conditions can modify, rather than eliminate, UV-B-induced physiological inhibition through potential buffering mechanisms.

BSCs at different developmental stages exhibited distinct response strategies. In this study, the physiological plasticity of algal crusts was relatively limited following UV-B stress, whereas lichen and moss crusts exhibited stronger dynamic regulatory capacity and recovery potential. Redundancy analysis further indicated that UV-B radiation was the dominant factor driving physiological and metabolic differentiation in BSCs (F = 39.53, P = 0.001), while crust type and the warming and reduced precipitation treatment also played important regulatory roles.

Overall, the results of this two-year experiment demonstrate that predictions of BSC responses to future climate change should incorporate developmental-stage heterogeneity and interactions among multiple climatic drivers. However, it should be noted that the warming and reduced precipitation conditions in this study were generated simultaneously by OTCs; therefore, the observed physiological and metabolic responses represent the combined effects of these environmental changes rather than the independent effects of warming or precipitation reduction alone. Future studies using factorial experimental designs, including independent warming and precipitation manipulation treatments, are needed to disentangle the individual and interactive effects of these climate drivers on BSC physiological and metabolic processes. Furthermore, investigations across broader climatic gradients, longer temporal scales, and diverse BSC communities are needed to determine the generality of these stage-dependent response strategies.

## Data Availability

The raw data supporting the conclusions of this article will be made available by the authors, without undue reservation.
