# Supplementary material for: Photosynthetic and secondary metabolic responses of biological soil crusts to combined warming, reduced precipitation, and enhanced UV-B radiation: divergent strategies across developmental stages
Source: Front Plant Sci. 2026 Sep 9;17:1930890. doi: 10.3389/fpls.2026.1930890 (PMC13597266; doi:10.3389/fpls.2026.1930890)
Supplement: Supplementary file 1 [file Table1.docx]

| Response Variable | Normality | Homogeneity of variance | Transformation | Final method |
| --- | --- | --- | --- | --- |
| Chl a | Passed | Passed | None | Three-way ANOVA |
| Chl b | Passed | Passed | None | Three-way ANOVA |
| GPP | Passed | Passed | None | Three-way ANOVA |
| Flavonoid | Passed | Passed | None | Three-way ANOVA |
| Total phenolic | Passed | Passed | None | Three-way ANOVA |
| Car | Failed after transformation | Passed | Transformation attempted but assumptions not satisfied | ART-ANOVA |
| EPS | normal after log transformation | Passed | log transformation | Three-way ANOVA |

**Supplementary Table 1.** Statistical methods used for each response variable.

Normality was tested using the Shapiro-Wilk test, and homogeneity of variance was tested using Levene’s test. Variables satisfying parametric assumptions after transformation were analyzed using conventional three-way ANOVA. Variables that still failed to meet parametric assumptions were analyzed using ART-ANOVA.
